# Research progress of Traditional Chinese Medicine (TCM) in targeting inflammation and lipid metabolism disorder for arteriosclerosis intervention: A review

**DOI:** 10.1097/MD.0000000000033748

**Published:** 2023-05-05

**Authors:** Xiaoyu Xuan, Jingyi Zhang, Jilin Fan, Shiliang Zhang

**Affiliations:** a First School of Clinical Medicine, Shandong University of Traditional Chinese Medicine, Jinan, China; b Department of Cardiology, The Affiliated Hospital of Shandong University of Traditional Chinese Medicine, Jinan, China.

**Keywords:** atherosclerosis, foam cells, inflammation, Traditional Chinese medicine

## Abstract

Atherosclerosis (AS) is a chronic disease caused by inflammation and lipid deposition. Immune cells are extensively activated in the lesions, producing excessive pro-inflammatory cytokines, which accompany the entire pathological process of AS. In addition, the accumulation of lipid-mediated lipoproteins under the arterial intima is a crucial event in the development of AS, leading to vascular inflammation. Improving lipid metabolism disorders and inhibiting inflammatory reactions are the primary treatment methods currently used in medical practice to delay AS progression. With the development of traditional Chinese medicine (TCM), more mechanisms of action of the monomer of TCM, Chinese patent medicine, and compound prescription have been studied and explored. Research has shown that some Chinese medicines can participate in treating AS by targeting and improving lipid metabolism disorders and inhibiting inflammatory reactions. This review explores the research on Chinese herbal monomers, compound Chinese medicines, and formulae that improve lipid metabolism disorders and inhibit inflammatory reactions to provide new supplements for treating AS.

## 1. Introduction

Cardiovascular disease (CVD) is the leading cause of death in the global population.^[1,2]^ It poses a severe threat to the health and life of patients and increases the economic burden on society and families. Research shows that in 2019, approximately 18.5 million people died globally due to cardiovascular-related diseases, accounting for 1-third of all deaths, of which over 1.2 million occurred in individuals under 50.^[3]^ Atherosclerosis (AS) is the primary pathological basis for CVD, which manifests clinically as ischemic heart disease, stroke, and peripheral vascular disease, among others.^[4]^ The occurrence of AS is related to various risk factors, including hypercholesterolemia, hypertension, diabetes, smoking, obesity, dietary habits, age, gender, genetics, and work-life stress.^[5–10]^

As the main pathogenesis of AS, lipid metabolism disorders, inflammatory reactions intersect and affect the occurrence and development of AS on multiple levels. When LDL continuously accumulates under the endothelium, it is oxidized into oxidized low-density lipoprotein (oxLDL) through lipid peroxidation induced by free radicals. These oxidized lipids can interact with immune and endothelial cells and actively participate in the inflammatory process (Supplementary Fig. 1, http://links.lww.com/MD/I978).^[11]^ oxLDL is a potent inducer of inflammation and can be recognized by TLRs to trigger inflammatory signals, induce the expression of transcription factor NF-kB, and increase the release of pro-inflammatory cytokines, promoting the recruitment of immune cells to the intima.^[12]^ Among them, macrophages can take up oxLDL and transform it into foam cells. Foam cells can secrete various cytokines (such as IL-1 and IL-6) to amplify the inflammatory response locally. The NLRP3 inflammasome is a multiprotein complex that can activate the abundant presence of oxLDL and cholesterol crystals in atherosclerotic lesions. Therefore, NLRP3 links lipid deposition in the vascular wall with inflammatory reactions.^[13]^

In addition, most lipid-lowering drugs have anti-inflammatory and immune-regulatory effects. For example, studies have shown that statins can have anti-inflammatory effects by improving vascular risk factors. The above studies show the complex relationship between lipid metabolism and inflammation.^[14]^ On the other hand, innate and adaptive immunity can also regulate lipid metabolism. Inflammation can reduce the expression of several lipases through multiple pathways. For example, when TLRs are activated, they can inhibit the expression of ABCA1 mediated by liver X receptor (LXR), reducing cholesterol efflux.^[15]^ Although lipid-lowering drugs have effective anti-inflammatory effects, the blood lipid-lowering effect of the drugs was insignificant. Therefore, the accumulation of cholesterol can activate the cascade of inflammatory signals to induce an inflammatory response. In contrast, activating inflammatory reactions can damage lipid hydrolysis and efflux, increasing cholesterol accumulation. Ultimately, both factors jointly affect the pathological process of AS.

Traditional Chinese medicine (TCM) has a long history and significant efficacy in treating AS. Single herbs, Chinese patent medicines, and compound formulas can intervene in AS by targeting inflammation and lipid metabolism disorders.^[16]^ Curcumin, quercetin, and resveratrol are TCM most widely used natural components in treating CVD. As a drug for the prevention and treatment of AS, quercetin has been shown to have anti-inflammatory, antioxidant, and endothelial protective effects. Quercetin anti-inflammatory effect is achieved by reducing the expression level of inflammatory factors by blocking the STAT-3 pathway. Meanwhile, quercetin can also inhibit lipid deposition induced by oxLDL.^[17]^ Studies have shown that combining aspirin and compound Danshen dripping pills have better clinical efficacy in treating coronary heart disease than aspirin alone.^[18]^

Most of the references cited in this study come from relevant literature reports from PubMed and Web of Science in the past 20 years. The search keywords include “atherosclerosis,” “TCM,” “Traditional Chinese medicine,” “Chinese patent medicine,” “compound prescription,” “inflammation,” and “foam cells” et al This review aims to introduce the efficacy of Chinese medicine in clinical applications by discussing the inflammatory lipid mechanisms underlying AS and the latest research progress on Chinese medicine treatment of AS.

## 2. TCM in the treatment of arteriosclerosis

**Figure 1.**
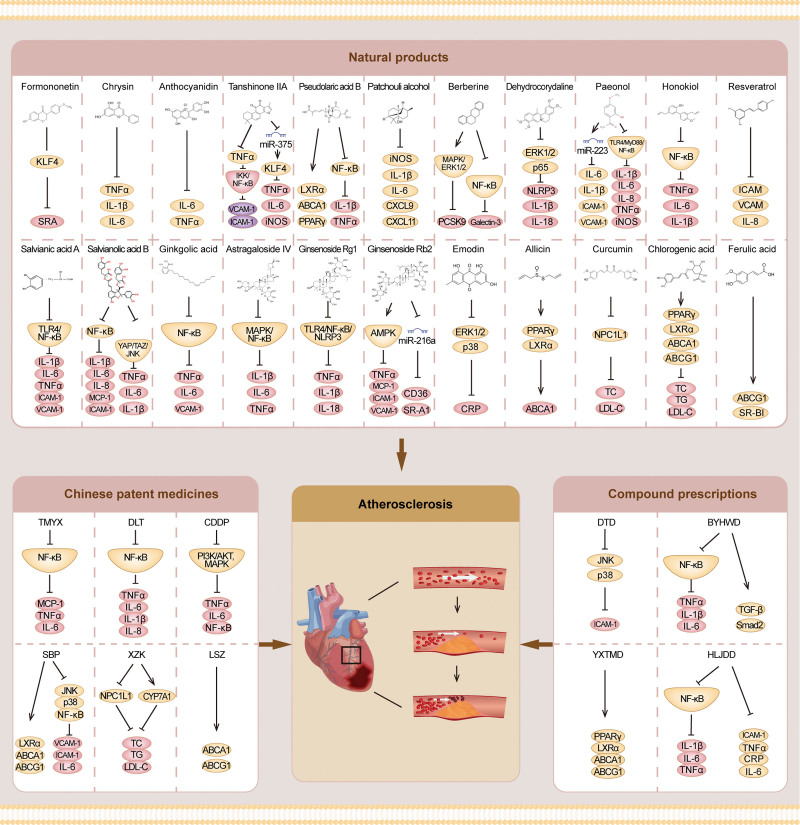
Diagram of the main signaling pathways leading to the development of atherosclerosis.

Multiple active ingredients of natural medicine possess cardiovascular benefits, including flavonoids, terpenoids, phenylpropanoids, alkaloids, and quinonoid, which can give rise to anti-arteriosclerotic effects via intervention on arteriosclerotic inflammation, accumulation of lipids, metabolism of endothelial cells and macrophages and the proliferation and migration of cells. The following section discusses the therapeutic effect of TCM monomers, which are represented by flavonoids, terpenoids, and alkaloids (Supplementary Table S1, http://links.lww.com/MD/I979). Moreover, the evidence and mechanism of traditional Chinese patent medicines, simple preparations (Supplementary Table S2, http://links.lww.com/MD/I980), and compound prescriptions (Supplementary Table S3, http://links.lww.com/MD/I981) in the treatment of AS are elaborated through examples (Fig. 1).^[19,20]^ This study summarizes data from published articles and does not address issues related to patient ethics, etc. Therefore, this study does not require approval by the ethics committee.

### 2.1. Inhibiting the inflammatory response

Functional disorders of endothelial cells are a significant pathological characteristic of early AS. When endothelial cells lining blood vessels are impaired, it facilitates the infiltration of oxLDL into the sub-endothelial layer. Consequently, substantial adhesion factors attract white blood cells and monocytes to the vascular wall and incite a local inflammatory reaction. This process ultimately leads to the formation of atherosclerotic plaques in the affected area. TCM, such as natural monomers, traditional Chinese patents, and compound prescriptions, can treat AS by inhibiting the release of inflammatory factors from relevant immune cells. Thus, we summarize and analyze the detailed mechanism of some herbal medicines that reduce and inhibit inflammation, which helps treat AS.

#### 2.1.1. Natural monomers

##### 2.1.1.1. Flavonoids

Herbs containing flavonoids can regulate blood lipids by inhibiting vascular inflammation response and suppressing the progression of AS. The activity of neutrophils is closely associated with inflammation. Studies have shown that apigenin, luteolin, and wogonin can induce neutrophil time- and concentration-dependent apoptosis, thereby preventing persistent inflammatory. Flavonoids have the potential to become a new generation of anti-inflammatory compounds.^[21]^ Ma et al discovered that formononetin extracted from TCM milkvetch root, can inhibit monocyte adherence to endothelial cells and reduce inflammation in ApoE-deficient mice model.^[22]^ In a randomized, double-blind, placebo-controlled cross-trial, 37 (pre) hypertensive male and female patients were studied to determine the effect of epicatechin and quercetin on endothelial dysfunction and biomarkers of inflammation. The study suggested that epicatechin can protect the heart by improving endothelial function, while quercetin can inhibit inflammation.^[23]^ However, a study^[24]^ showed contrasting results, demonstrating that administering quercetin to patients with post-myocardial infarction significantly enhanced their total antioxidant capacity but did not significantly affect inflammatory factors. Tahereh Farkhondeh et al^[25]^ found that chrysin, a flavonoid more abundant in propolis, can inhibit inflammation in aged rats that were administered intraperitoneal injections, thereby inhibiting hyperlipidemia, hyperglycemia, and obesity. This study involved 169 individuals with blood lipid abnormalities randomly assigned to either a placebo group or different doses of anthocyanins (40 mg/day, 80 mg/day, and 320 mg/day). The results indicated that anthocyanidin exhibit anti-inflammatory and antioxidant effects that depend on the dosage. An intake of 80mg/day of anthocyanidin has been shown to reduce inflammation and oxidative stress, with a more pronounced effect observed with 320 mg/day. Therefore, taking more than 80 mg of anthocyanidin per day could lower CVD risk.^[26–28]^

##### 2.1.1.2. Terpenoids

Terpenoid compounds are commonly found worldwide and are an essential active ingredient in certain medical plants with anti-inflammation pharmacological function. Tanshinone IIA derived from the dried roots of Salvia Miltiorrhiza, has been shown to protect the cardiovascular system through multiple mechanisms. It can inhibit the expression of TNF-α, which degrades cellular adhesion molecules and chemotactic factors.^[29]^ Moreover, A study applied Tanshinone IIA to male ApoE^−\−^ mice fed a high-fat diet. The results showed it might elicit anti-inflammatory action and stabilize the plaques through the TLR4/myeloid differentiation primary response 88/NF-κB signaling pathway.^[30]^ TLR4 and TLR2 are the 2 most important members of the TLR family. Based on the 2 primary adaptor proteins involved in TLR signaling, the pathway can be divided into 2 general pathways, the myeloid differentiation primary response 88-dependent and adapter-inducing interferon-β (TRIF) -dependent pathways. Research indicates that obesity, as a cardiovascular risk factor, can worsen the progression of AS by enhancing TLRs stimulation and leading to higher levels of pro-inflammatory cytokines.^[31]^ NF-κB is a critical transcription factor that can enhance the expression of various inflammatory cytokines and play an essential role in immune and inflammatory responses. Activation the NF-κB signaling pathway is crucial for inflammation induced by multiple factors such as cytokines, adhesion factors, chemotactic factors, growth factors, and monocytes attached to endothelial cells.^[32]^ Pseudolaric acid B (PB) is a diterpene acid extracted from the roots and bark of Pseudolarix kaempferi Gorden. It can markedly suppress the activity of Ly6Chi inflammatory monocytes in peripheral blood and potentially alleviate inflammation mediated by macrophages in a PPAR-γ-dependent manner.^[33]^ Patchouli alcohol (PA) is a tricyclic sesquiterpene extracted from the cablin patchouli herb. Research indicates that PA can reduce experimental animal AS by inhibiting macrophage infiltration and its inflammatory response. Wang et al conducted a study in which high-fat-fed ApoE^−\−^ mice were randomly divided into PA and non-treatment groups. The study found that the PA group reduced cytokines mRNA expression, including MCP-1, IL-1β, and IL-6 while inhibiting macrophages inflammation. These findings suggest that PA may alleviate AS without affecting lipid metabolism.^[34]^

##### 2.1.1.3. Alkaloids

Alkaloids are primary nitrogenous essential compounds that primarily exist in plants. *Berberine* is an alkaloid extracted from Coptidis Rhizoma. By using Western blotting and confocal microscopy, it has been found that berberine can downregulate the expression of Galectin-3, an inflammatory regulator mainly expressed in macrophage. This is achieved through inhibition of the NF-κB signaling pathway and activation of the AMPK signaling pathway, ultimately alleviating inflammation.^[35]^ Rutaecarpine is a promising alkaloid known for its cardioprotective properties due to its cardiotonic, anti-inflammatory, and anti-oxidative effects and ability to regulate monocytes and macrophages.^[36]^ Luo et al conducted an in vitro and in vivo experiment to investigate the anti-atherosclerotic effects and mechanisms of 5-deoxy-rutaecarpine (R3), a derivative of rutaecarpine. It has been demonstrated that R3 can alleviate AS by inhibiting NLRP3inflammasome-related inflammation and regulating cholesterol transportation.^[37]^ NLRP3 is one of the most extensively studied members of the NLR family. Studies have shown that cholesterol crystals can activate the NLRP3 inflammasome, leading to an inflammatory response and promoting the formation of AS. Notably, the absence of key components of the NLRP3 inflammasome has been shown to reduce the severity of AS and inflammation significantly.^[38]^ Thus, rutaecarpine can exert anti-atherosclerotic effects by regulating the NLRP3 inflammasome. Dehydrocorydaline (DHC), one of the main active alkaloids in Yanhusuo, a TCM, is mainly used in clinical practice to treat coronary heart disease and acute myocardial infarction, as well as to alleviate inflammatory pain. Research reports indicate that DHC has anti-inflammatory properties and can effectively alleviate inflammatory pain. Consequently, it is widely used to treat spasmodic pain, abdominal pain, and pain resulting from injuries.^[39]^ However, research on the role of DHC in AS remains relatively limited. Nonetheless, studies have demonstrated that intraperitoneal injection of DHC in ApoE^−/−^ mice can inhibit AS progression and stabilize plaques. In addition, DHC can alleviate systemic vascular inflammation by inhibiting the activation of p65 and extracellular signal-regulated kinases 1/2 in macrophages.^[40]^ Therefore, the anti-inflammatory properties of DHC in the occurrence and development of AS warrant further investigation to elucidate its mechanisms.

#### 2.1.2. Traditional Chinese patent medicines and simple preparations

Naoxintong (NXT) Capsules contain herbal ingredients, including Astragalus, Angelica sinensis, red peony root, Pheretima, Sichuan lovage rhizome, Carthamus tinctorius L, and Peach seed et al When used alone or conjunction with conventional intervention measures, NXT has been shown to effectively treat CVD without causing severe adverse events. Derived from Buyang Huanwu Decoction (BYHWD), NXT capsules have been found to reduce levels of TNF-α and P-selectin in serum and to inhibit the expression of inflammatory molecules, thus exhibiting anti-inflammatory and antioxidative properties.^[41]^ Simultaneous intake of NXT with black false hellebore or TCM containing similar components to black false hellebore is not advised. Additionally, NXT should not be used by individuals with bleeding tendencies, menstruating women, or those undergoing anticoagulant or antiplatelet therapies.^[42]^ Tongmai Yangxin (TMYX) pill contains ingredients like Polygonum multiflorum, Radix Ophiopogonis, Liquorice, Caulis Spatholobi, and Rehmannia, is commonly used to treat coronary heart disease, angina, and arrhythmia. Tan et al conducted a study measuring serum biochemical markers, including apoB, endothelin 1, and NF-κB, in patients with coronary heart disease after 8 weeks TMYX treatment. The results showed a significant decrease in these markers. In addition, an in vitro study evaluated the anti-inflammatory effect of ethanol extract from TMYX using Murine cell RAW264.7. The results indicated that TMYX anti-inflammatory activity of TMYX regulates Estrogen receptor 1 and NF-κB signaling pathway activity.^[43]^ TMYX can be used as an adjuvant therapy for coronary artery atherosclerotic heart disease, but it is not a mainstream drug. Danlou Tablets (DLT) is an alternative therapy for treating angina pectoris in coronary heart disease patients experiencing chest tightness, chest pain, and breath-holding symptoms. A study on the ApoE^−/−^ mice model treated with DLT therapy for 4 weeks, with a daily dosage of 700 mg/kg, showed improvements in heart structure, tissue pathology, and AS plaque. The study suggests that DLT anti-AS mechanism may be partially by regulating the NF-κB signaling pathway.^[44]^

#### 2.1.3. Compound prescription

Compound prescriptions in TCM have been found to intervene in inflammation and TCM monomers. For example, a study collected Daotan Decoction orally to rats for 3 days and collected their serum to observe its effects on TNF-α-stimulated HUVECs. The results showed that Daotan Decoction inhibited the expression of ICAM-1 by inhibiting the activation of JNK and p38.^[45]^ Liu et al employed network pharmacology and experimental validation methods to demonstrate that BYHWD could suppress inflammatory cytokines and block the NF-κB signaling pathway.^[46]^ Another study used network pharmacology and clinical experiments to explore the anti-inflammatory effects of the core components of Zhishi Xiebai Guizhi Decoction. The mechanism may be closely related to PPARγ, inflammation, TNF signaling pathway, AMPK signaling pathway, and PI3K-Akt signaling pathway.^[47]^

### 2.2. Reduction of the accumulation of lipids and formation of foam cells

The formation of foam cells is an early event in the development of AS. When low-density lipoprotein penetrates the intima of the artery and enters the space between the blood vessel walls, cholesterol accumulates in the wall. When cholesterol accumulates to a certain extent, the endothelial cells of the blood vessel release hormones to attract monocytes and trigger their differentiation into macrophages. Macrophages engulf oxidized cholesterol produced by themselves and try to digest the fat. The fat accumulated in the macrophages makes them foam cells. The accumulation of foam cells forms lipid stripes and plaques, ultimately leading to the development of AS. Research shows that effective components in TCM can slow the formation of atherosclerotic plaques by inhibiting the foam cell formation of macrophages, reducing the expression of inflammatory factors, and lowering lipid peroxidation reactions. In the following, we report the studies on inhibiting foam cell formation and the degradation process of some herbal medicines.

#### 2.2.1. Natural monomers

##### 2.2.1.1. Flavonoids

One study indicated that formononetin might decrease the expression of SRA, thus reducing the production and accumulation of foam cells derived from VSMCs and macrophages.^[22]^ A randomized, double-blind, placebo-controlled trial assigned 374 patients to a baicalin or placebo group. After 12 weeks, the baicalin group exhibited significant improvements in indices such as triglycerides, total cholesterol, and LDL compared to the placebo group.^[48]^ In an experiment, oxLDL was administered to culture RAW264.7 macrophages to establish a foam cell model. Treatment with quercetin reduced lipid accumulation and delayed aging, which has been associated with macrophage autophagy.^[49]^

##### 2.2.1.2. Terpenoids

Li et al utilized ApoE^−/−^ mice fed with a high-fat diet and RAW264.7 macrophages as in vivo and in vitro models, respectively, to investigate the anti-atherosclerotic effects of PB and its molecular mechanism.^[33]^ The result showed that PB could reduce the levels of TC, TG, and LDL-C, inhibit the uptake of macrophage lipids by promoting the expression of LXRα, ABCA1, and PPARγ, and promote the efflux of cholesterol by inhibiting the activation of NF-κB in ApoE^−/−^ mice.^[33]^ Lectin-like oxidized low-density lipoprotein receptor-1 (LOX-1), a scavenger receptor, can mediate the cell to recognize and take in oxLDL. Moreover, tanshinone IIA, when used in vitro to treat cultured mouse macrophages, was found to decrease LOX-1 expression by inhibiting the NF-kB signaling pathway, which, in turn, reduced lipid intake and foam cell accumulation.^[50]^ Furthermore, another study showed that Tanshinone IIA could reduce foam cells accumulation by strengthening macrophage autophagy, thereby delaying AS progression.^[51]^

##### 2.2.1.3. Alkaloids

In ApoE^−/−^ mice subjected to a high-fat diet, berberine has been shown to reduce plasma lipid level by regulating the PI3K/AKT/mTOR signaling pathway and autophagy, thereby countering lipids accumulation in the carotid artery.^[52]^ Tan et al treated ApoE^−/−^ mice with berberine and explored its anti-atherosclerotic effect mechanism using TMT-based proteomics and bioinformatics techniques.^[53]^ The study showed that berberine treatment significantly lowered oxLDL, TG, and FFA levels, countered hepatic lipid accumulation, improves intima-media thickening, and alleviates atherosclerotic lesions.^[53]^ Additionally, another study^[54]^ indicated that berberine improved lipid profiles and hepatic fat accumulation, potentially through the down-regulation of PCSK9 mediated by the MAPK/extracellular signal-regulated kinases 1/2 signal pathway. Clinical trials involving patients with hyperlipidemia showed that berberine effectively reduced TC and potentially LDL-C with no noticeable adverse effects.^[55]^ Another clinical trial involving 144 low-risk CVD subjects who took berberine 500 mg twice a day for 3 months demonstrated a decrease in TC, TG, and LDL-C and an increase in HDL-C concentration. Therefore, the above research shows that the berberine clinical application may be a promising treatment method for improving high blood lipid levels in patients with CVD.^[56]^

#### 2.2.2. Traditional Chinese patent medicines and simple preparations

Xuezhikang capsules, an extract of red yeast rice used to treat hyperlipidemia, effectively regulate cholesterol homeostasis. Isoflavones and phytosterols present in the capsules have been shown to improve the elimination of bile acid and reduce cholesterol absorption in the intestinal tract of mice fed a high-fat diet.^[57]^ Longshengzhi Capsules derived from BYHWD have significantly reduced aorta lesions induced by a high-fat diet in ApoE^−/−^ mice. This is achieved by activating the ABCA1/ABCG1, downgrading the gene expression, associated with adipogenesis and cholesterol synthesis, and exhibiting a potent anti-inflammatory effect.^[58]^ Hedan Tablet is a commonly used traditional Chinese patent medicine that has been found to contain Nuciferine, Tanshinone I1A, Salvianic acid A, Ursolic acid, Sennoside A and B, Psoralen, and Angelicin. This medicine effectively reduces cholesterol, triglycerides, and body weight, while increasing the concentration of high-density lipoprotein, improving lipid metabolism disorders, and reducing the incidence of atherosclerotic plaques. In a study involving 37 patients with hyperlipidemia showed that Hedan Tablet slightly reduced the plasma level of LDL after administering 4.38 g/day for 8 consecutive weeks, exhibiting favorable effects on HDL subfractions.^[59]^ Tongxinluo Capsule, a widely used TCM, could reduce lipid accumulation by enhancing Beclin-1-induced autophagy and promoting the autophagic outflow of lipids.^[60]^ In a study involving 1212 patients with carotid intima-media thickness (IMT) ≥ 1.2 mm, the treatment group continued to take Tongxinluo Capsules for 24 months. Compared with the placebo group, the treatment group showed delayed progression of mean intima-media thickness, plaque area, and vascular remodeling of the carotid artery.^[61]^

#### 2.2.3. Compound prescriptions

The PPARγ-LXRα-ABCA1/ABCG1 pathway shows promise as a new target for treating AS due to its essential role in regulating cholesterol efflux. However, the use of PPARγ agonists in AS treatment remains controversial due to the observed systemic adverse reactions in some patients. Zheng et al found that Yinxing Tongmai Decoction could attenuate AS in ApoE^−/−^ mice, by activating the PPARγ-LXRα-ABCA1\ABCG1 pathway to strengthen efflux of cholesterol, thus improving the inflammation microenvironment.^[62]^ Zhang et al established an AS model in high-fat-fed ApoE^−/−^ mice and treated them with Dingxin Recipe IV for 12 weeks. Their results show that Dingxin Recipe Ⅳ can attenuate AS by reducing the accumulation of lipids and regulating the level of TG, TC, LDL-C, and HDL-C via the LXR-α\SREBP1 pathway to regulate lipid metabolism.^[63]^ The modified Tongmai Zhuyu Tang (TMZYT) has a similar therapeutic effect as atorvastatin, and the combination of both produces a better result.^[64]^ Moreover, BYHWD can significantly reduce the lipid content in the aortic sinus, lower the levels of TC, TG, and LDL-C in serum, ameliorate hyperlipidemia, regulate lipid metabolism, and retard plaque progression.^[65]^

## 3. Discussion

AS is a widespread vascular disease, and antioxidant therapy is crucial in its treatment. However, the clinical efficacy of available antioxidants is currently limited. The accumulation of reactive oxygen species (ROS) produced from cell oxidation-reduction reaction can lead to structural damage. These ROS are primarily produced within cell organelles such as the mitochondrial inner membrane and peroxisomes. The human body has evolved 2 antioxidant defense systems to counteract ROS, 1 endogenous and the other exogenous. The human body can synthesize endogenous (enzymatic and non-enzymatic) antioxidants, while exogenous antioxidants must be supplemented through diet. Therefore, Chinese medicine mainly uses natural plant components to provide exogenous antioxidants, which have therapeutic effects on AS. Studies have shown that natural polyphenolic compounds derived from plants, fruits, and natural extracts can effectively treat inflammation, aging, cancer, and CVD by regulating the mitochondrial biogenesis signaling pathway.^[66]^

The pharmacological effects of TCM often stem from a complex composition. However, modern scientific technology in clinical research about TCM provides a more scientific approach, enabling its inherent characteristics and clinical therapeutic value to be fully realized. In recent years, numerous studies have explored the relevance of TCM and its extracts in biomedical and food applications.^[67–69]^ As a natural, nontoxic, and environmentally friendly reducing agent, the extraction process of Euphorbia leaf can reduce silver ions on the inner surface of urea-based periodic mesoporous organosilica to silver nanoparticles, thereby preventing the aggregation of nanoparticles, reducing toxicity, and enhancing antimicrobial properties.^[70,71]^ Due to its good antibacterial effect, it is often used as a candidate material to reduce hospital infections.^[72,73]^ The combination of nanotechnology and TCM makes up for its shortcomings in clinical applications, improves its bioavailability and therapeutic targeting, prolongs circulation time, and enhances therapeutic effects.^[74]^ Flavonoids are widely distributed in angiosperms, with more in plants such as Rutaceae, Asteraceae, and Leguminosae.^[75]^

In medicine, flavonoids are primarily utilized for developing available drugs, including UV protectants,^[76]^ antivirals,^[77]^ and treatments for cardiovascular and cerebrovascular diseases.^[78]^ Numerous studies have demonstrated the positive effects of flavonoids on human health, which can be applied to developing various health products for the human body, offering new possibilities in the food industry.^[79]^ Flavonoids also possess significant therapeutic effects in medicine, such as their ability to combat CVD, anti-inflammatory, antipyretic, analgesic, anti-swelling, antibacterial, antiviral, and antioxidant free radicals.^[80–82]^ In a study, synthesized surface-modified Astragalus Polysaccharides were synthesized and loaded onto selenium nanoparticles containing Tanshinone IIA.^[83]^ The study improved the bioavailability of both components.^[84]^ It fully exerted its antioxidant capabilities, inhibiting the production of ROS, providing a new method for antioxidant therapy for spinal cord injury. Therefore, flavonoids can be an effective supplement for treating CVD in the medical field. With the continuous research and development of terpenoid essential oils in recent years, more plant essential oils are used to treat CVD.^[85]^ Terpenoid compounds related to CVD are mainly limonene and saponin. Limonene is a monocyclic terpene abundant in citrus fruit peel oil. Isovalene compounds, abundant in food seasonings, fragrances, wine, and some plant oils, can synthesize limonene. Saponins are glycosides of triterpenes, and soy saponins are the most effective in influencing lipid metabolism.^[86]^ Experimental studies have shown that soy saponins can inhibit lipid oxidation in serum, inhibit the generation of peroxidized lipids, and reduce the content of cholesterol and triglycerides in the blood.^[87]^ Studies have also shown that soy saponins can inhibit fibrinogen and platelet aggregation, regulate the body hemolytic system, and have anti-thrombotic effects.^[88]^ In addition, soy saponins can reduce oxygen utilization, improve myocardial oxygen supply, and enhance the body hypoxia tolerance.^[89]^ In studies of notoginsenoside-based biological preparations, notoginsenoside R1-loaded mesoporous silica nanoparticles can improve heart function by targeting damaged myocardium after myocardial infarction.^[90]^ Plants of the Mentha genus are rich in essential oils and phenolic compounds. Because they are natural, their antioxidant and antibacterial activities make them safe alternatives for synthetic preservatives to extend the shelf life of fruits, vegetables, and meat products.^[90–93]^

The latest research shows that plant nanoparticles have emerged as a promising new avenue for treating AS. Yuyu Li et al^[94]^ have successfully synthesized modified macrophage membrane-coated nanoparticles capable of transporting colchicine to atherosclerotic sites. These nanoparticles exhibit excellent targeting endothelial cells in the inflammatory environment while evading macrophage phagocytosis, thus offering a potential treatment for AS. Similarly, Kim H et al^[95]^ has developed cyclodextrin-statin self-assembled drug complex nanoparticles using cyclodextrin and statin drugs to treat AS. The synergistic effect of cyclodextrin and statin drugs can enhance drug delivery and anti-inflammatory effects targeting plaques. Cyclodextrin causes cholesterol regression in established plaques, and statin drugs can inhibit the proliferation of macrophage foam cells, thereby achieving the therapeutic goal of AS. Sean Marrache^[96]^ and his team directed macrophages to track apoptosis by detecting the collapse of mitochondrial membrane potential during cell death using constructed nanoparticles. In vitro experiments showed that cells quickly removed the nanoparticles and could detect apoptosis and bind to cholesterol. They believe that the developed nanomaterials have the potential to serve as carriers, aiding in the early diagnosis of AS and prevention of vulnerable plaque formation. Therefore, the correlation research on constructing nanoparticles with effective components of TCM will provide prospects for treating AS.

The mechanisms behind the formation of arteriosclerosis are complex, which poses a challenge for traditional natural drugs to treat AS effectively. First, additional research and clinical trials are necessary to fully comprehend these mechanisms and overcome the unspecific anti-arteriosclerotic molecular mechanisms present in Chinese herbs. Second, conducting more rigorous clinical trials is essential to provide robust evidence for clinical use. Most current studies have been limited to in vitro testing, with only a few clinical trials conducted. Third, the efficacy of the ingredients used in Chinese Material Medica is uncertain, as not all active ingredients have been specified. Because the vast majority of TCM and their preserved formulas are based on the experience and summary of Chinese people in treating diseases for over 2000 years, it is not necessarily that one or several components play a genuine therapeutic effect during the treatment of a particular disease. However, some trace components that have yet to be reported and studied may play the most critical role. Due to considerations of protecting their product patents and interests, some TCM manufacturers may keep the actual formula of the medicine confidential or not fully disclose the effective components of the medicine in the instructions. Accordingly, different research methods applied in the studies above may cause contradictory results even though the examined Chinese material medica is the same.

To improve the clinical application of TCM, it is necessary to conduct further scientific extraction and separation of ingredients, determine the active ingredient, and give more attention to drug toxicity and adverse reactions. Additionally, developing safer and more effective dosage forms is crucial. Currently, research mainly focuses on the efficacy of ingredients while giving less attention to side effects and low oral bioavailability of certain ingredients. With advancements in extraction and separation techniques and the scope of research, it is possible to elaborate on the active ingredients and mechanisms of action, providing novel therapeutic strategies and molecular targets for arteriosclerosis.

## 4. Conclusion

This study comprehensively summarizes the detailed mechanisms by which TCM regulates inflammatory responses and foam cell metabolism in treating AS. The article discusses how monomers, Chinese patent medicines, and Chinese herbal compound prescriptions extracted from traditional herbs inhibit the expression of pro-inflammatory cytokines such as IL-1β and IL-6 by inhibiting signaling pathways such as NF-κB and JAK/STAT, ultimately preventing AS. Additionally, they regulate cholesterol intake and outflow, reduce foam cell aggregation, and regulate lipid metabolism, ultimately improving AS. This review summarizes the research progress on treating AS with TCM, clarifies the effectiveness of clinical applications and provides evidence to support the broader use of TCM.

## Acknowledgments

XX and JZ were in charge of searching all the relative papers and writing this manuscript. JF was in charge of drawing the picture. SZ gave valuable and professional suggestions and guidance in organizing and drafting this manuscript.

## Author contributions

**Conceptualization:** Xiaoyu Xuan, Shiliang Zhang.

Data curation: Xiaoyu Xuan.

Formal analysis: Jingyi Zhang.

Funding acquisition: Shiliang Zhang.

Investigation: Jingyi Zhang.

Methodology: Jingyi Zhang.

Project administration: Shiliang Zhang.

Resources: Jingyi Zhang.

Software: Jilin Fan.

Supervision: Jilin Fan.

Validation: Jilin Fan.

Visualization: Jilin Fan.

Writing – original draft: Xiaoyu Xuan.

Writing – review & editing: Shiliang Zhang.

## Supplementary Material








