# Supplementary figures and images for: Research progress of Traditional Chinese Medicine (TCM) in targeting inflammation and lipid metabolism disorder for arteriosclerosis intervention: A review
Source: Medicine (Baltimore). 2023 May 5;102(18):e33748. doi: 10.1097/MD.0000000000033748 (PMC10158879; doi:10.1097/MD.0000000000033748)

**Supplementary Figure 1.** Immune responses in atherosclerosis.

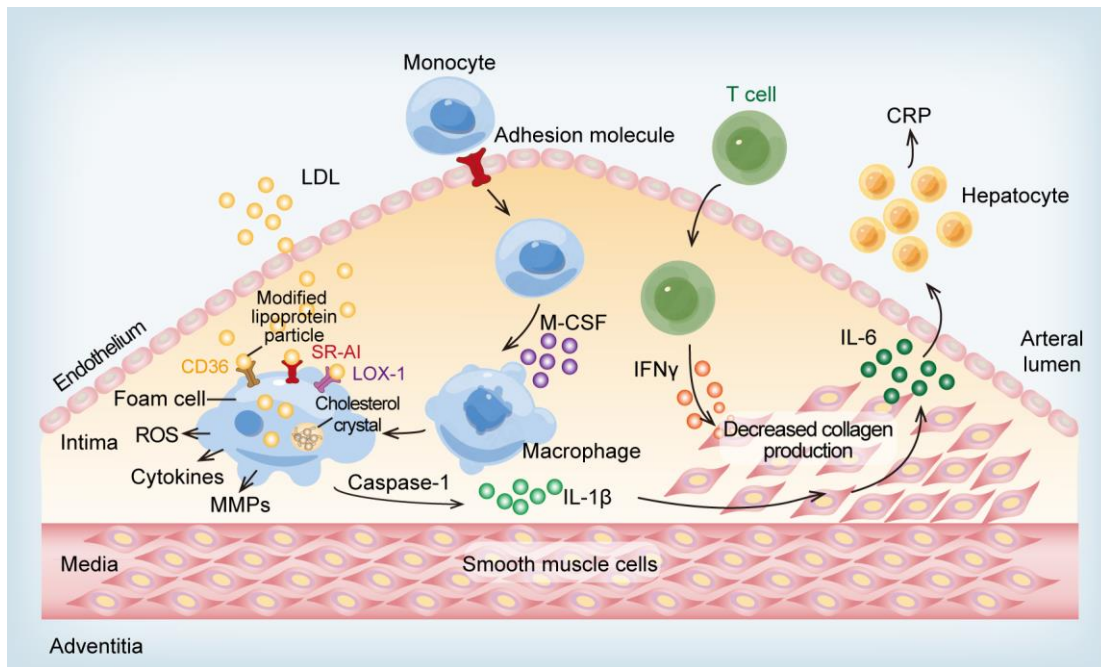

Supplement: Supplementary file 1 [file medi-102-e33748-s001.pdf]
