# Supplementary material for: Research progress of Traditional Chinese Medicine (TCM) in targeting inflammation and lipid metabolism disorder for arteriosclerosis intervention: A review
Source: Medicine (Baltimore). 2023 May 5;102(18):e33748. doi: 10.1097/MD.0000000000033748 (PMC10158879; doi:10.1097/MD.0000000000033748)
Supplement: Supplementary file 2 [file medi-102-e33748-s002.pdf]

Supplementary Table S1. Important anti-atherosclerotic traditional Chinese medicine monomers.

**Active Ingredients**	**Source**	**Structure**	**Experimental Model**	**Efficacy**	**Mechanism**	**Refs**
Apigenin, luteolin, Wogonin	Apium graveolens, Mignonette, Radix Scutellariae	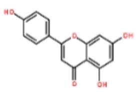<br>Apigenin<br>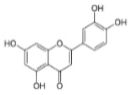<br>luteolin<br>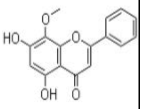<br>Wogonin	Granulocytes; Transgenic zebrafish	Induces neutrophil apoptosis and resolves inflammation.	In a caspase-dependent fashion to override survival factor-induced delay of apoptosis; down-regulates the key neutrophil survival protein Mcl-1 in a proteasomal-dependent fashion.	1
Formononetin	Astragalus	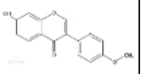	ApoE$^{-/-}$mice	Reduces face and aortic root sinus lesions size; Reduces foam cell formation.	Decreases SRA expression and reduces monocyte adhesion; KLF4 negatively regulates the expression of SRA.	2
Quercetin, Epicatechin	Quercus dentata, Green Tea.	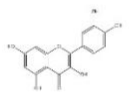<br>Quercetin<br>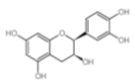<br>Epicatechin	Thirty-seven (pre)hypertensive men and women (40–80 y).	Improves endothelial function and reduces inflammation.	Reduces sE-selectin; Reduces IL-1β and the z score for inflammation.	3
Quercetin	Quercus dentata	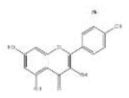	88 post-MI patients	Elevates TAC and improves the insecurity dimension of QOL.	Increases serum TAC;lowers the scores of insecurity; No significant changes in IL-6, hs-CRP, blood pressure and other QOL dimensions.	4
			RAW264.7 macrophages treated with ox-LDL.	Inhibits foam cell formation; delays senescence.	Regulates MST1-mediated autophagy of RAW264.7 cells.	5
Chrysin	Propolis; Oroxylum indicum	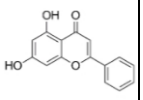	Male Wistar rats (2, 10, and 20 month-old)	Inhibits hyperlipidemia, hyperglycemia, and obesity.	Attenuates age-related lipid abnormalities, glucose elevation, and inflammation.	6

Anthocyanidin	Lycuum ruthenicum Murr	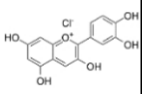	169 participants with dyslipidemia	Anti-oxidative and anti-inflammatory.	Not shown.	7
Tanshinone II A	Salvia Miltiorrhiza	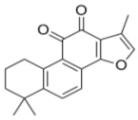	HUVECs treated with Tan II A	Down-regulation of cell adhesion molecules and chemokine expression.	Inhibition of TNF-α-induced NF-κB activation and IκBα phosphorylation by the suppression of the IKK pathway.	8
			Male ApoE$^{-/-}$ mice (6 weeks)	Anti-Inflammatory and Immune-Regulating effects.	Via the TLR4/MyD88/ NF-κB signaling pathway.	9
			RAW264.7 macrophages treated with ox-LDL.	Inhibits foam cell formation.	Reduces the expression of scavenger receptor LOX-1 via inactivating NF-kB signaling pathway.	10
			ApoE$^{-/-}$mice	Enhances autophagy and M2 polarization of Macrophage.	Activates KLF4 by inhibiting miR-375.	11
Pseudolaric acid B (PB)	Pseudolarix kaempferi Gorden	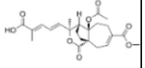	Male ApoE$^{-/-}$ mice; RAW264.7 macrophage.	Attenuates atherosclerosis progression and inflammation.	Suppresses PPARγ-mediated NF-κB activation; Promotes the expression of LXRα, ABCA1 and PPARγ.	12
Patchouli alcohol	Pogostemonis herba	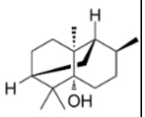	ApoE$^{-/-}$mice	Significantly attenuates atherosclerotic plaque burdens in both the aorta and the aortic root.	Significantly reduces the mRNA expression of proinflammatory iNOS, IL-1β, IL-6, CXCL9 and CXCL1.	13
Berberine	Coptidis Rhizoma	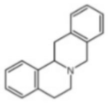	THP- 1- derived macrophages were pretreated with BBR	Alleviates ox-LDL-induced macrophage activation.	Downregulates galectin- 3 via the NF- κB and AMPK signaling pathways.	14
			ApoE$^{-/-}$mice	Reduces serum lipid levels; improves intimal hyperplasia; antagonizes carotid lipid accumulation.	Regulates the PI3K/AKT/mTOR signaling pathway; regulates autophagy; promotes cell proliferation and inhibits cell apoptosis.	15
			ApoE$^{-/-}$mice	Reduces serum lipid levels, antagonizes hepatic lipid accumulation, improves intima-media thickening, and	Regulation of mitochondrial dysfunction and targeting of APOA1.	16

				alleviates atherosclerotic lesions.		
			ApoE$^{-/-}$mice	Ameliorates the extent of HFD-induced atherosclerosis, improves the lipid profiles and hepatic fat accumulation.	Down-regulation of PCSK9 mediated by the MAPK/ERK1/2 signal pathway.	17
5-deoxy-rutaecarpine (R3)	Laboratory synthesis	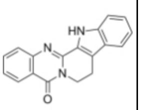 Euodia rutaecarpa	ApoE$^{-/-}$mice	Attenuates atherosclerosis development and increases plaque stability; Decreases levels of inflammatory mediators.	Inhibits NLRP3 inflammasome activation by inhibiting NF-κB and MAPK pathways; Decreases total cholesterol through upregulating protein expression of ABCA1 and SR-B1/human CD36.	18
Dehydrocorydaline	Corydalis yanhusuo	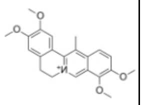	ApoE$^{-/-}$mice; Bone marrow-derived macrophages (BMDMs).	Attenuates atherosclerosis; Reduces systemic and vascular inflammation.	Inhibits inflammation, likely by targeting macrophage p65- and ERK1/2-mediated pathways.	19
Baicalin	Radix Scutellariae	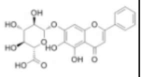	Three hundred 74 patients with CAD and RA.	Reduces blood lipids and inflammation.	Not shown.	20

Reference

1. Lucas CD, Allen KC, Dorward DA, et al. Flavones induce neutrophil apoptosis by down-regulation of Mcl-1 via a proteasomal-dependent pathway. *FASEB J.* 2013;27(3):1084-1094.
2. Ma C, Xia R, Yang S, et al. Formononetin attenuates atherosclerosis via regulating interaction between KLF4 and SRA in apoE(-/-) mice. *Theranostics.* 2020;10(3):1090-1106.
3. Dower JI, Geleijnse JM, Gijsbers L, Schalkwijk C, Kromhout D, Hollman PC. Supplementation of the Pure Flavonoids Epicatechin and Quercetin Affects Some Biomarkers of Endothelial Dysfunction and Inflammation in (Pre)Hypertensive Adults: A Randomized Double-Blind, Placebo-Controlled, Crossover Trial. *J Nutr.* 2015;145(7):1459-1463.
4. Dehghani F, Sezavar Seyedi Jandaghi SH, Janani L, Sarebanhassanabadi M, Emamat H, Vafa M. Effects of quercetin supplementation on inflammatory factors and quality of life in post-myocardial infarction patients: A double blind, placebo-controlled, randomized clinical trial. *Phytother Res.* 2021;35(4):2085-2098.
5. Cao H, Jia Q, Yan L, Chen C, Xing S, Shen D. Quercetin Suppresses the Progression of Atherosclerosis by Regulating MST1-Mediated Autophagy in ox-LDL-Induced RAW264.7 Macrophage Foam Cells. *Int J Mol Sci.* 2019;20(23):6093.

6. Farkhondeh T, Abedi F, Samarghandian S. Chrysin attenuates inflammatory and metabolic disorder indices in aged male rat. *Biomed Pharmacother.* 2019;109:1120-1125.
7. Zhang H, Xu Z, Zhao H, et al. Anthocyanin supplementation improves anti-oxidative and anti-inflammatory capacity in a dose-response manner in subjects with dyslipidemia. *Redox Biol.* 2020;32:101474.
8. Chang CC, Chu CF, Wang CN, et al. The anti-atherosclerotic effect of tanshinone IIA is associated with the inhibition of TNF-alpha-induced VCAM-1, ICAM-1 and CX3CL1 expression. *Phytomedicine.* 2014;21(3):207-216.
9. Chen Z, Gao X, Jiao Y, et al. Tanshinone IIA Exerts Anti-Inflammatory and Immune-Regulating Effects on Vulnerable Atherosclerotic Plaque Partially via the TLR4/MyD88/NF-kappaB Signal Pathway. *Front Pharmacol.* 2019;10:850.
10. Xu S, Liu Z, Huang Y, et al. Tanshinone II-A inhibits oxidized LDL-induced LOX-1 expression in macrophages by reducing intracellular superoxide radical generation and NF-kappaB activation. *Transl Res.* 2012;160(2):114-124.
11. Chen W, Li X, Guo S, et al. Tanshinone IIA harmonizes the crosstalk of autophagy and polarization in macrophages via miR-375/KLF4 pathway to attenuate atherosclerosis. *Int Immunopharmacol.* 2019;70:486-497.
12. Li T, Wang W, Li YX, et al. Pseudolaric acid B attenuates atherosclerosis progression and inflammation by suppressing PPARgamma-mediated NF-kappaB activation. *Int Immunopharmacol.* 2018;59:76-85.
13. Wang HT, Wang ZZ, Wang ZC, et al. Patchouli alcohol attenuates experimental atherosclerosis via inhibiting macrophage infiltration and its inflammatory responses. *Biomed Pharmacother.* 2016;83:930-935.
14. Pei C, Zhang Y, Wang P, et al. Berberine alleviates oxidized low-density lipoprotein-induced macrophage activation by downregulating galectin-3 via the NF-kappaB and AMPK signaling pathways. *Phytother Res.* 2019;33(2):294-308.
15. Song T, Chen WD. Berberine inhibited carotid atherosclerosis through PI3K/AKTmTOR signaling pathway. *Bioengineered.* 2021;12(1):8135-8146.
16. Tan W, Wang Y, Wang K, et al. Improvement of Endothelial Dysfunction of Berberine in Atherosclerotic Mice and Mechanism Exploring through TMT-Based Proteomics. *Oxid Med Cell Longev.* 2020;2020:8683404.
17. Ma CY, Shi XY, Wu YR, et al. Berberine attenuates atherosclerotic lesions and hepatic steatosis in ApoE(-/-) mice by down-regulating PCSK9 via ERK1/2 pathway. *Ann Transl Med.* 2021;9(20):1517.
18. Luo J, Wang X, Jiang X, et al. Rutaecarpine derivative R3 attenuates atherosclerosis via inhibiting NLRP3 inflammasome-related inflammation and modulating cholesterol transport. *FASEB J.* 2020;34(1):1398-1411.
19. Wen B, Dang YY, Wu SH, et al. Antiatherosclerotic effect of dehydrocorydaline on ApoE(-/-) mice: inhibition of macrophage inflammation. *Acta Pharmacol Sin.* 2022;43(6):1408-1418.
20. Hang Y, Qin X, Ren T, Cao J. Baicalin reduces blood lipids and inflammation in patients with coronary artery disease and rheumatoid arthritis: a randomized, double-blind, placebo-controlled trial. *Lipids Health Dis.* 2018;17(1):146.
