# Supplementary material for: Research progress of Traditional Chinese Medicine (TCM) in targeting inflammation and lipid metabolism disorder for arteriosclerosis intervention: A review
Source: Medicine (Baltimore). 2023 May 5;102(18):e33748. doi: 10.1097/MD.0000000000033748 (PMC10158879; doi:10.1097/MD.0000000000033748)
Supplement: Supplementary file 3 [file medi-102-e33748-s003.pdf]

Supplementary Table S2. Important anti-atherosclerotic traditional Chinese medicine.

**Chinese patent medicine**	**Experimental Model**	**Efficacy**	**Mechanism**	**Refs**
Tongmai Yangxin Pill	CHD patients	Attenuates macrophage foam cell formation and exhibits anti-inflammatory activity.	Regulates ESR1 and NF-κB signaling pathway activity.	1
Danlou tablet	$ApoE^{-/-}$mice	Regulates the levels of TNF-α, IL-6, IL-1β, IL-8, MMP-1 and MMP-2, as well as protein expression of NF-κB p-50 and IκB-α, and promotes cell expression of NF-κB p-50, IκB-α and phospho-NF-κB p-50.	Regulates of NF-κB signaling pathway.	2
Xuezhikang capsule	Six-week-old specific-pathogen-free (SPF) male C57BL/6 mice	Regulates cholesterol homeostasis.	Enhances the elimination of bile acids and reduces intestinal cholesterol absorption.	3
LongShengZhi Capsule	$ApoE^{-/-}$mice	Ameliorates hepatic lipid metabolism and inhibits inflammation.	Reduces macrophage/foam cell accumulation by activating ABCA1/ABCG1 expression; Down-regulates expression of lipogenic and cholesterol synthetic genes while activating expression of triglyceride catabolism genes; Reduces serum TNF-αlevels, infiltration of neutrophils, Kupffer cells, and expression of inflammatory cytokines in the liver.	4
Hedan Tablet	Patients with hyperlipidemia.	Decreases the levels of LDL-C and has a favorable modification of the HDL subfraction distribution.	Not shown.	5

Reference

1. Fan Y, Liu J, Miao J, et al. Anti-inflammatory activity of the Tongmai Yangxin pill in the treatment of coronary heart disease is associated with estrogen receptor and NF-kappaB signaling pathway. *J Ethnopharmacol.* 2021;276:114106.

2. Gao S, Xue X, Yin J, et al. Danlou tablet inhibits the inflammatory reaction of high-fat diet-induced atherosclerosis in ApoE knockout mice with myocardial ischemia via the NF-kappaB signaling pathway. *J Ethnopharmacol.* 2020;263:113158.
3. Feng D, Sun JG, Sun RB, et al. Isoflavones and phytosterols contained in Xuezhikang capsules modulate cholesterol homeostasis in high-fat diet mice. *Acta Pharmacol Sin.* 2015;36(12):1462-1472.
4. Ma J, Zhao D, Wang X, et al. LongShengZhi Capsule Reduces Established Atherosclerotic Lesions in apoE-Deficient Mice by Ameliorating Hepatic Lipid Metabolism and Inhibiting Inflammation. *J Cardiovasc Pharmacol.* 2019;73(2):105-117.
5. Xu RX, Wu NQ, Li S, et al. Effects of Hedan Tablet () on lipid profile, proprotein convertase subtilisin/kexin type 9 and high-density lipoprotein subfractions in patients with hyperlipidemia: A primary study. *Chin J Integr Med.* 2016;22(9):660-665.
