# Supplementary material for: Research progress of Traditional Chinese Medicine (TCM) in targeting inflammation and lipid metabolism disorder for arteriosclerosis intervention: A review
Source: Medicine (Baltimore). 2023 May 5;102(18):e33748. doi: 10.1097/MD.0000000000033748 (PMC10158879; doi:10.1097/MD.0000000000033748)
Supplement: Supplementary file 4 [file medi-102-e33748-s004.pdf]

Supplementary Table S3. Important anti-atherosclerotic compound prescriptions.

Compound	Experimental Model	Efficacy	Mechanism	Refs
Dao Tan decoction	Male Sprague-Dawley rats; HUVECs treated with TNF-α.	Inhibits TNF-α-induced ICAM-1 expression.	Blocks JNK and p38 signaling pathways.	1
BuYangHuanWu decoction	Male Sprague-Dawley (SD) rats are fed with high fat diet.	Decreases the inflammation, lipid level; improves the morphology of the aortic intima.	Blocks NF-κB signaling pathways	2
Zhishi Xiebai Guizhi Decoction	Patients with coronary heart disease.	Anti-inflammatory effects.	May be relate to PPARγ, TNF signaling pathway, AMPK signaling pathway, and PI3K-Akt signaling pathway.	3
Yinxing Tongmai decoction	$ApoE^{-/-}$mice	Inhibits foam cell formation.	Activates the PPARγ-LXRα-ABCA1/ABCG1 pathway to enhance cholesterol efflux.	4
Dingxin Recipe IV	$ApoE^{-/-}$mice	Inhibits cholesterol deposition in aorta; Regulates the level of TG, TC, LDL-C, and HDL-C.	Through LXR-α/SREBP1 pathway.	5
Tongmai Zhuyu Decoction	Male Wistar rats; C57BL/6J mice.	Attenuates aortic intima thickening and foam cell accumulation; Decreases the inflammation, lipid level.	Through modulation gut microbiota.	6

Reference

1. Huang X, Wang F, Chen W, et al. Dao-Tan decoction inhibits tumor necrosis factor-alpha-induced intercellular adhesion molecule-1 expression by blocking JNK and p38 signaling pathways in human umbilical vein endothelial cells. *Pharm Biol.* 2012;50(9):1111-1117.
2. Liu B, Song Z, Yu J, Li P, Tang Y, Ge J. The atherosclerosis-ameliorating effects and molecular mechanisms of BuYangHuanWu decoction. *Biomed Pharmacother.* 2020;123:109664.
3. Gao J, Pan Y, Zhao Y, et al. Network Pharmacology Study on Molecular Mechanisms of Zhishi Xiebai Guizhi Decoction in the Treatment of Coronary Heart Disease. *Evid Based Complement Alternat Med.* 2021;2021:3574321.
4. Zheng S, Huang H, Li Y, et al. Yin-xing-tong-mai decoction attenuates atherosclerosis via activating PPARgamma-LXRalpha-ABCA1/ABCG1 pathway. *Pharmacol Res.* 2021;169:105639.

5. Zhang Y, Gu Y, Chen Y, et al. Dingxin Recipe IV attenuates atherosclerosis by regulating lipid metabolism through LXR-alpha/SREBP1 pathway and modulating the gut microbiota in ApoE(-/-) mice fed with HFD. *J Ethnopharmacol.* 2021;266:113436.
6. Ji W, Jiang T, Sun Z, et al. The Enhanced Pharmacological Effects of Modified Traditional Chinese Medicine in Attenuation of Atherosclerosis Is Driven by Modulation of Gut Microbiota. *Front Pharmacol.* 2020;11:546589.
